# Atypical femoral fracture: The periprosthetic variant about two cases without bisphosphonate use

**DOI:** 10.1051/sicotj/2026038

**Published:** 2026-07-03

**Authors:** Guillaume Auberger, Thomas Aubert, Younes Kerroumi, Philippe Leclerc, Simon Marmor

**Affiliations:** Diaconesses Croix Saint-Simon Hospital Group 125 rue d’Avron 75020 Paris France

**Keywords:** Periprosthetic fracture, Atypical femoral fracture, AFF

## Abstract

Atypical femoral fractures are rare. They are well described in patients with prolonged bisphosphonate use. The periprosthetic form without bisphosphonate intake appears to be exceptional and may lead to mechanical complications (nonunion, secondary displacement) if the difficulties of bone healing are not anticipated. We report here two cases of atypical femoral fractures without bisphosphonate use and the resulting implications for surgical fixation.

## Introduction

Although rare, atypical femoral fractures (AFFs) pose a real challenge for both diagnosis and medical-surgical management. The American Society for Bone and Mineral Research (ASBMR) has long described major and minor criteria to aid in the diagnosis of such fractures (Table 1).

**Table 1:** (4 major criteria needed) Diagnostic criteria for atypical femoral fractures.

Major criteria	Minor criteria
– Fracture is associated with minimal or no trauma, as in a fall from a standing height or less.	– Generalized increase in cortical thickness of the femoral diaphyses.
– Fracture line originates at the lateral cortex and is substantially transverse in its orientation, although it may become oblique as it progresses medially across the femur.	– Unilateral or bilateral prodromal symptoms such as dull or aching pain in the groin or thigh.
– Complete fractures extend through both cortices and may be associated with a medial spike; incomplete fractures involve only the lateral cortex.	– Bilateral incomplete or complete femoral diaphysis fractures.
– Fracture noncomminuted or minimally comminuted.	– Delayed fracture healing.
– Localized periosteal or endosteal thickening of the lateral cortex is present at the fracture site (“beaking” or “flaring”).	

The main causes of AFFs reported in the literature are the use of osteoclast inhibitors, particularly bisphosphonates and denosumab, usually after prolonged use, which alters bone remodeling.

The pathogenesis of AFFs remains poorly understood [1]. However, the literature consistently highlights a strong association with long-term bisphosphonate use.

We present two cases of female patients who met all diagnostic criteria for AFFs, but whose medical history did not include any causal medication. Both had unexplained hip prosthesis pain for several weeks. These cases raise questions about the pathophysiology of atypical femoral fractures and confirm the current recommendations regarding the need for rigid fixation strategies.

## Case presentations

### Case 1

An 86-year-old female was hospitalized for advanced bicompartmental knee osteoarthritis unresponsive to conservative treatment. She was 1.48 m tall and weighed 85 kg (BMI: 38 kg/m^2^). A total left knee replacement was indicated. Her medical history included:

- Restless legs syndrome.
- Right total hip arthroplasty for osteoarthritis (2022).
- Laminectomy L4–L5 (2022).
- Left femoral shaft fracture, treated by intramedullary nailing and later bone grafting (2020).

Her medications included pramipexole, gabapentin, and duloxetine.

Preoperatively, she reported contralateral thigh pain, which was initially managed conservatively (rest and analgesics).

**Figure 1:**
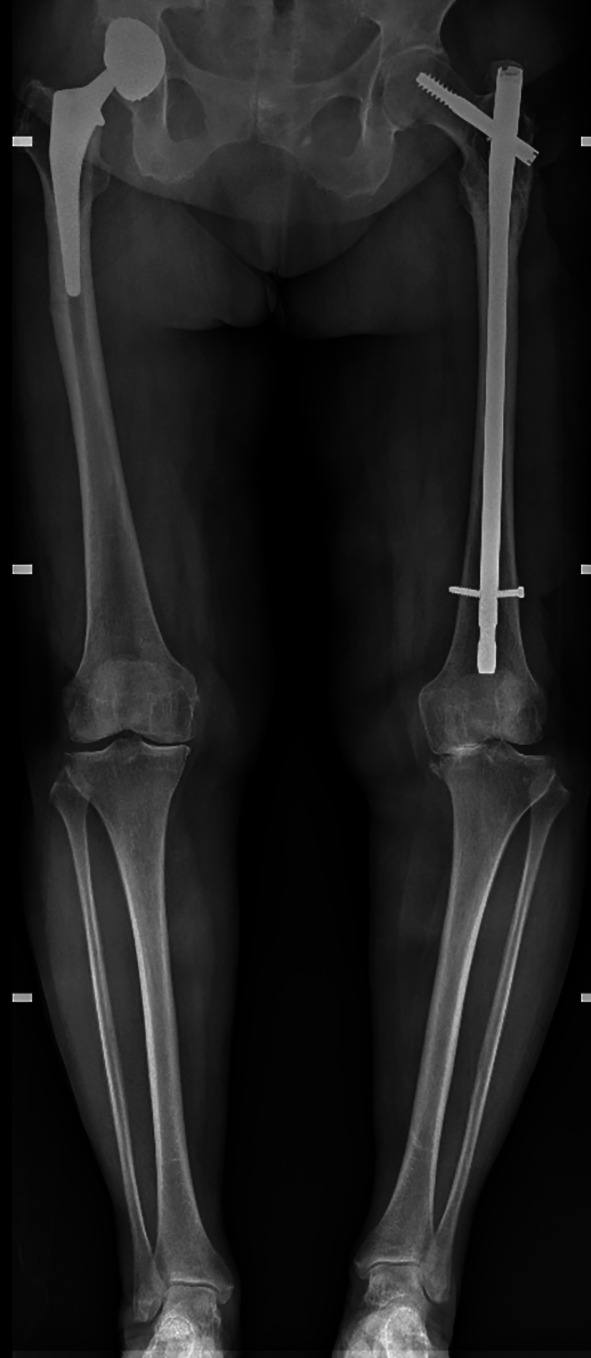
Incomplete atypical femoral fracture.

A left total knee arthroplasty (Attune®, Johnson & Johnson®) was performed with intraoperative navigation and kinematic alignment (Varus 12°–4°) (Figure 1). Postoperatively, she was mobilized but continued to experience contralateral thigh pain. She was discharged to a rehabilitation facility.

**Figure 2:**
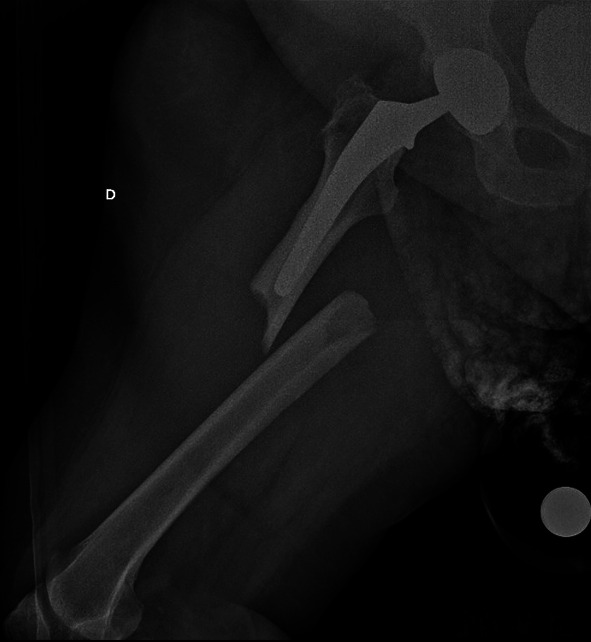
Complete atypical femoral fracture.

Shortly thereafter, the patient reported sudden functional impairment while sitting, without trauma. On readmission, radiographs revealed a right periprosthetic femoral fracture classified as AO/OTA 32A3, Vancouver C (Figure 2).

Pre-injury panoramic radiographs showed cortical thickening at the tip of the stem, followed by a stress fracture.

**Figure 3:**
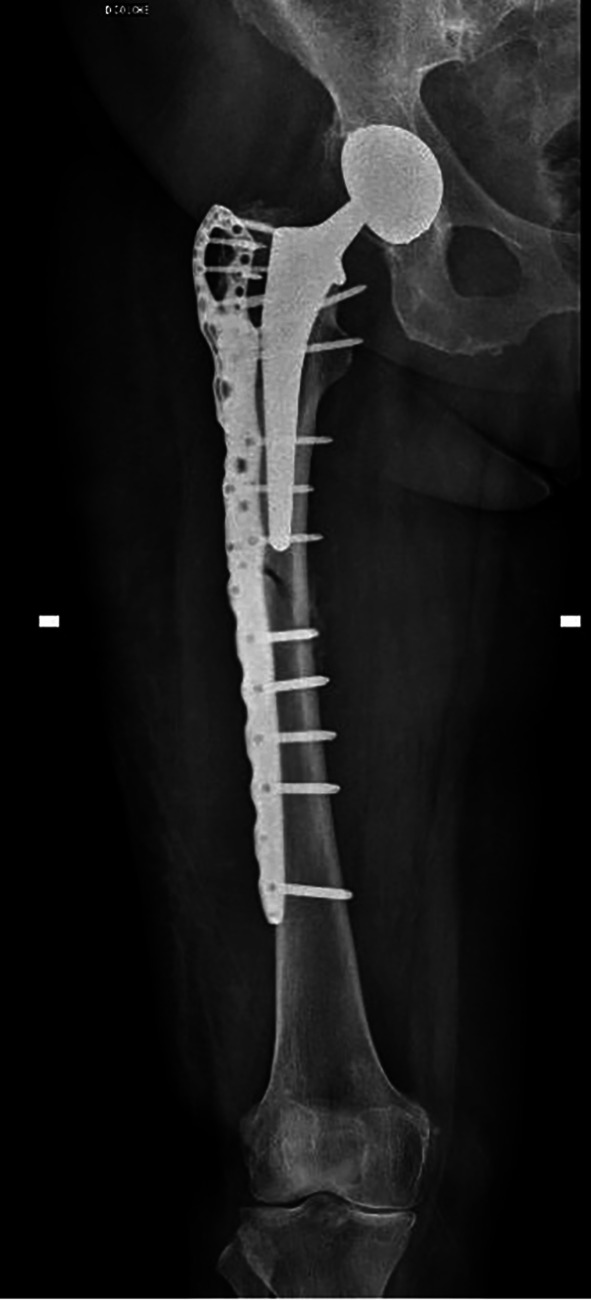
Internal fixation of the femoral fracture.

The patient underwent open reduction and internal fixation via a lateral subvastus approach using a Zimmer® NCB Periprosthetic plate with a 15 cm distal lever arm, 10-cortex screw fixation (distal plate screw density 0.83), and high-density proximal locking screws (Figure 3). Working length was nearly 3 cm.

She developed a postoperative pulmonary embolism, managed with curative anticoagulation (tinzaparin 14,000 IU anti-Xa/day, then apixaban 5 mg BID).

At 45 days post-op, full weight-bearing was authorized.

**Figure 4:**
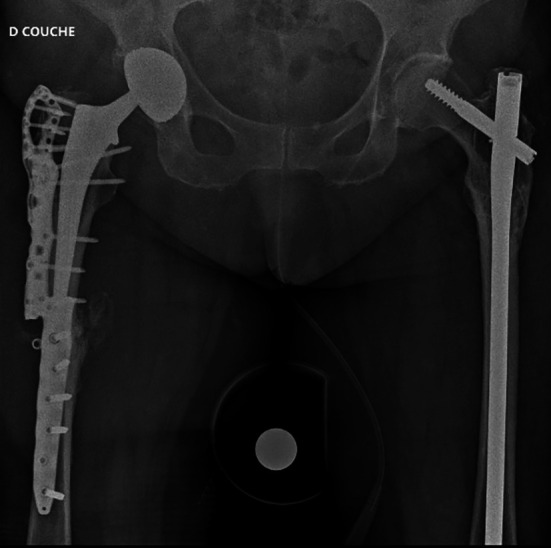
Iterative femoral Fracture and material breakage.

However, at day 82, she reported new groin and thigh pain without trauma. Imaging showed secondary displacement and plate failure (Figure 4).

**Figure 5:**
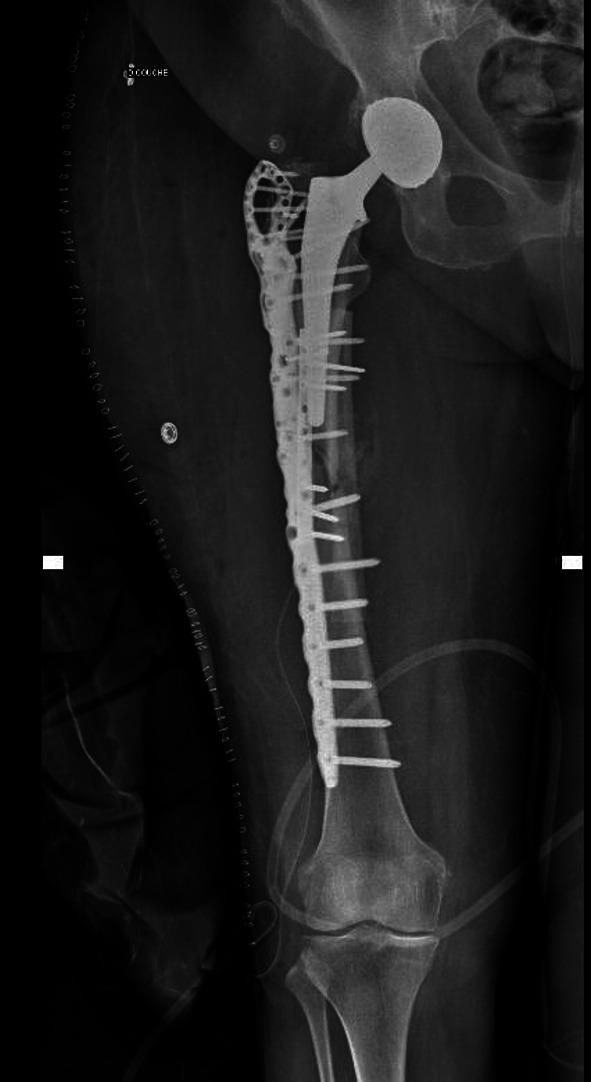
Iterative internal fixation enhanced with allograft.

She underwent revision surgery with a longer lateral plate, an anterior neutralization plate, and a posterior bone allograft (tibial strut) (Figure 5)

Histopathology from the fracture site revealed hypertrophic bone repair tissue with fibrous and cartilaginous components; no tumor or inflammation was found. Bacteriological cultures (3/3) were sterile.

**Figure 6:**
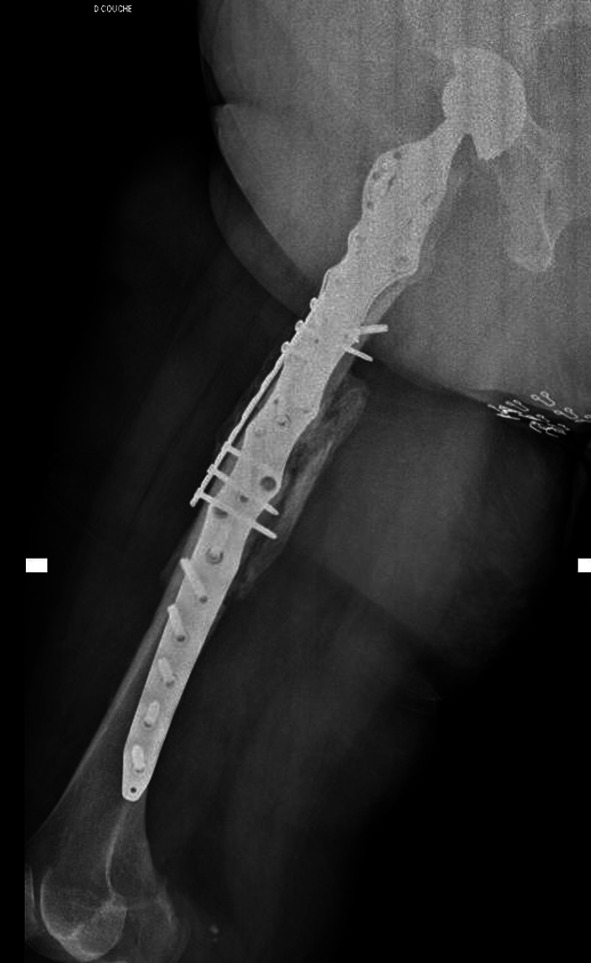
Posterior union on the lateral X-ray.

Postoperative protocol included toe-touch weight-bearing for 6 weeks. Bone healing was achieved at 6 months (Figure 6), and the patient resumed daily activities pain-free at the last follow-up (15 months).

### Case 2

Another 86-year-old female with a history of:

- Marfan syndrome.
- Breast cancer in remission.
- Left total knee arthroplasty.
- Bilateral total hip replacements for osteoarthritis (2010).

She measured 1.60 m, weighed 64 kg (BMI: 25 kg/m^2^), and her medications included prednisone, clopidogrel, and candesartan.

**Figure 7:**
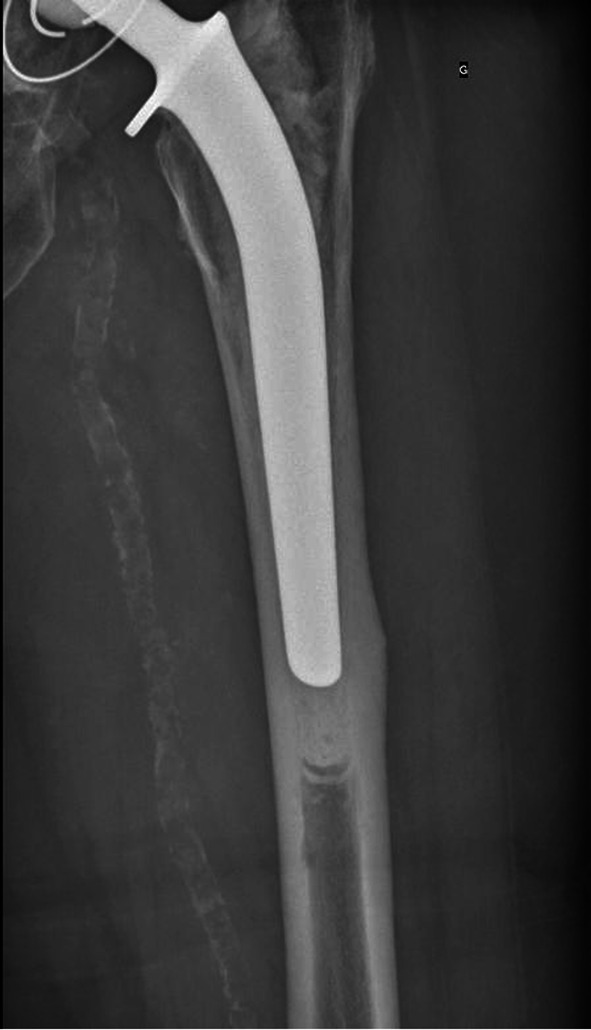
Incomplete atypical femoral fracture.

She consulted for persistent pain over her left hip prosthesis for 1 year. Radiographs were reported as normal (Figure 7), and a bone scan and articular injection were scheduled.

Seventeen days later, she returned with total functional impairment and visible thigh deformity without any traumatic event.

**Figure 8:**
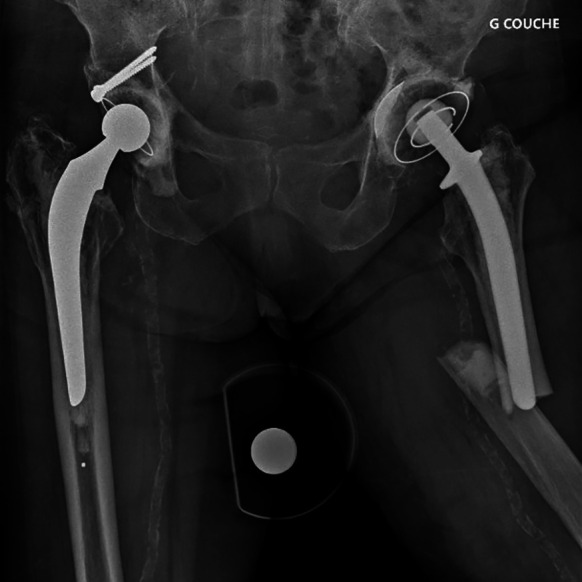
Complete atypical femoral fracture.

X-rays revealed a transverse left femoral fracture (AO/OTA 32A3) at the stem tip, Vancouver C (Figure 8).

**Figure 9:**
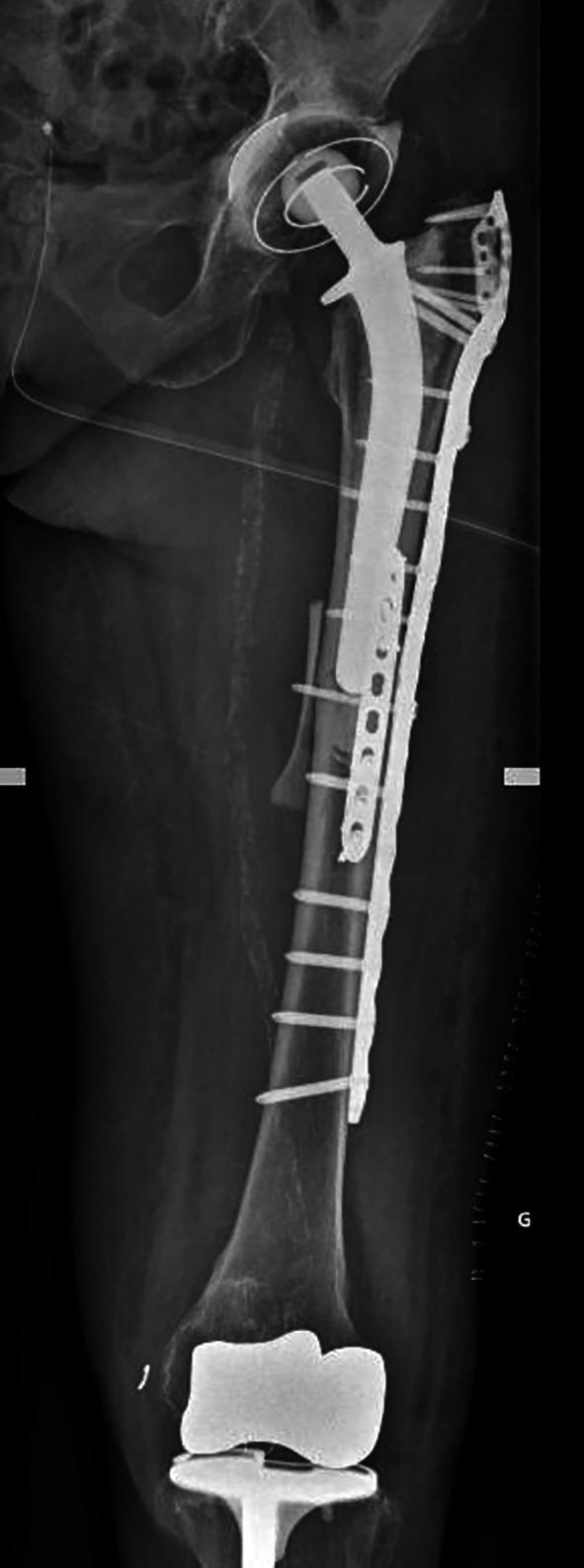
Primary internal fixation enhanced with allograft.

Given her clinical history, an allograft was planned upfront. Surgery was performed 2 days later: Open reduction and dual-plate fixation were performed with a Zimmer® NCB periprosthetic plate laterally (~20 cm distal lever arm; distal plate screw density 0.75), and a second orthogonal Synthes® plate. Posterior and medial strut allografts were added and secured with screws (Figure 9).

**Figure 10:**
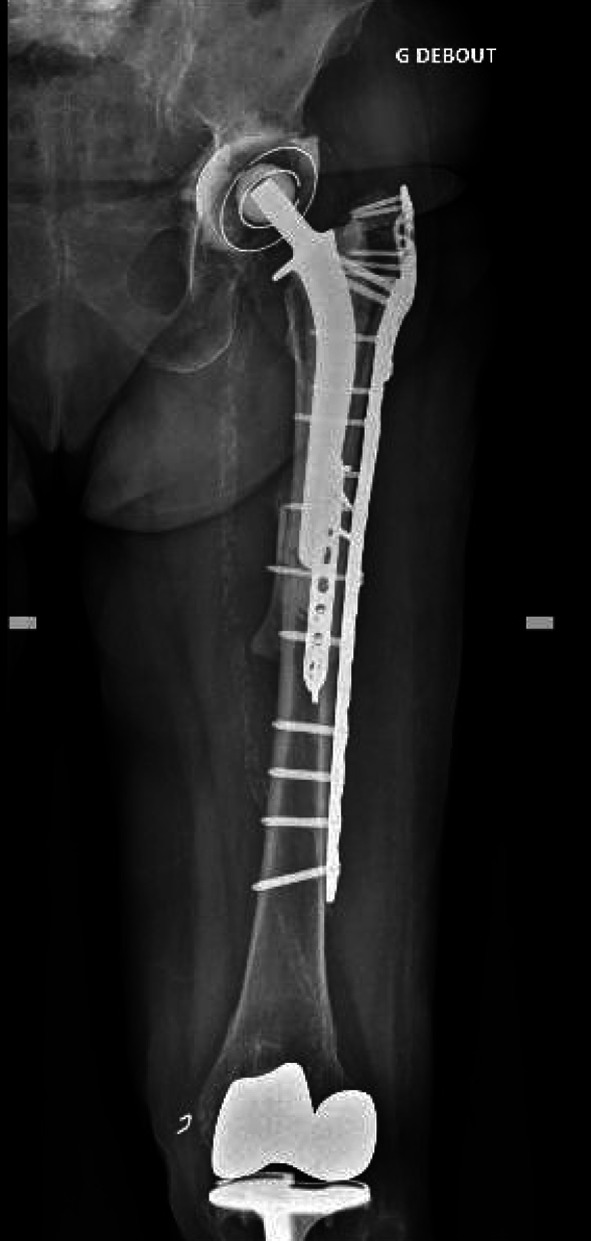
Union of the atypical femoral fracture.

She was discharged to a geriatric perioperative unit without complications. Toe-touch weight-bearing was permitted. At the 14-week follow-up, the patient was pain-free and allowed full weight-bearing. Radiographs showed early callus formation (Figure 10).

## Discussion

### Rare entity

Atypical femoral fractures are rare and poorly understood. More frequently reported in Asian populations, they are commonly associated with bisphosphonate therapy [2].

Periprosthetic AFFs have been described but are almost always linked to bisphosphonate use, as in a recent retrospective study of all periprosthetic fractures in a single center [3].

To our knowledge, this is the first case series of periprosthetic AFFs without any causative drug history.

Notably, in both cases, radiographs prior to the fracture were available.

### Pathophysiology

Bisphosphonates may compromise the femoral nutrient artery endothelium, generating a focal anterolateral stress concentration that can lead to fatigue fractures.

Analogous to the mechanics of felling a tree, where loading follows gravity lines, prosthetic stem tips may create a similar stress riser, particularly in curved femurs [4].

These fractures are low-energy, bending-type injuries, with no rotational component, explaining their classic non-comminuted morphology.

These two cases had a potential confounding factor. The first patient likely increased the load on the right lower limb due to the recent implantation of a contralateral knee prosthesis, and the second patient had Marfan syndrome and took corticotherapy, which can increase the risk of fracture.

### Management

#### Surgical treatment

AFFs are mechanically unstable and more prone to complications than typical femoral fractures [5], including nonunion and secondary displacement.

It is now established that fixation must be as rigid as possible (enhance the lever arm, diminish the working length, augment with an allograft). In the first case, the fixation used was a standard construct with 10 cortices on each side and a high screw density (83%), but without bone grafting. The rigidity of the construct was too low, and the fracture did not have enough time to heal before failing.

In native bone, intramedullary nails with multiple interlocking screws are preferred. For periprosthetic fractures, long plates with high-density screw fixation are required.

Adding an orthogonal second plate is often recommended for increased stability.

Allografts may enhance both mechanical strength (acting as a plate, allowing the sharing of load) and biological healing.

While replacing the stem with a long revision implant is a theoretical option, the risks of femoral osteotomy and implant-related complications make this a complex decision in periprosthetic cases.

#### Medical treatment

While bisphosphonates are implicated in AFFs, their role in preventing fragility fractures remains well-supported in the absence of contraindications.

Teriparatide has shown promise in enhancing the healing of AFFs but needs further high-quality studies.

Geriatric co-management improves postoperative outcomes in this frail population.

#### Contralateral and secondary prevention

Painful external cortical thickening, particularly at the stem tip, warrants consideration of preventive fixation.

A scoring system has been proposed to guide such decisions (indication if score ≥ 8) [6].

## Conclusion

This case series highlights a unique presentation of periprosthetic atypical femoral fractures without prior bisphosphonate use.

The recommended surgical strategy is rigid internal fixation, recognizing the typically slow healing process.

Painful prosthetic implants with external cortical thickening should prompt evaluation for preventive osteosynthesis.

## Data Availability

All the data are available.
